# A Healthful Plant-Based Diet as an Alternative Dietary Approach in the Management of Metabolic Dysfunction-Associated Steatotic Liver Disease

**DOI:** 10.3390/nu16132027

**Published:** 2024-06-26

**Authors:** Gabriele Castelnuovo, Nuria Perez-Diaz-del-Campo, Chiara Rosso, Angelo Armandi, Gian Paolo Caviglia, Elisabetta Bugianesi

**Affiliations:** 1 Department of Medical Sciences, University of Turin, 10126 Turin, Italy; gabriele.castelnuovo@unito.it (G.C.); nuria.perezdiazdelcampo@unito.it (N.P.-D.-d.-C.); chiara.rosso@unito.it (C.R.); angelo.armandi@unito.it (A.A.); gianpaolo.caviglia@unito.it (G.P.C.); 2 Gastroenterology Unit, Città della Salute e della Scienza—Molinette Hospital, 10126 Turin, Italy

**Keywords:** MASLD, steatosis, plant-based diet, lifestyle intervention and weight loss

## Abstract

Plant-based diets (PBDs) are gaining attention as a sustainable and health-conscious alternative for managing various chronic conditions, including metabolic dysfunction-associated steatotic liver disease (MASLD). In the absence of pharmacological treatments, exploring the potential of lifestyle modifications to improve biochemical and pathological outcomes becomes crucial. The adoption of PBDs has demonstrated beneficial effects such as weight control, increased metabolic health and improved coexisting diseases. Nonetheless, challenges persist, including adherence difficulties, ensuring nutritional adequacy, and addressing potential deficiencies. The aim of this review is to provide a comprehensive overview of the impact of PBDs on MASLD, emphasizing the need for tailored dietary interventions with professional support to optimize their effectiveness in preventing and treating metabolic diseases.

## 1. Introduction

Metabolic dysfunction-associated steatotic liver disease (MASLD), formerly known as non-alcoholic fatty liver disease, involves the presence of hepatic steatosis in combination with a cardiometabolic risk factor [1]. MASLD covers isolated hepatic steatosis, metabolic dysfunction-associated steatohepatitis (MASH), different degrees of liver fibrosis, cirrhosis and hepatocellular carcinoma (HCC) [2]. Hence, this new definition highlights the metabolic roots of the disease and its association with obesity, type 2 diabetes mellitus (T2DM) and other cardiometabolic risk factors [1,3]. The pathophysiology behind MASLD is complex, primarily driven by insulin resistance, leading to increased hepatic uptake of fatty acids and their accumulation in the liver [4,5]. Additionally, oxidative stress and chronic low-grade inflammation exacerbate liver damage, promoting progression from simple steatosis to more severe liver conditions [2]. This progression is often amplified by genetic predispositions and environmental factors, highlighting the need for targeted interventions [6].

The latest estimates indicate that the prevalence of the disease is increasingly reaching alarming levels, verging on a global pandemic [7]. MASLD is particularly common among individuals with obesity and T2DM, where the prevalence can increase two- to four-fold [8]. About one in four individuals with MASLD might develop MASH, which in the US is the second leading cause of liver transplants in adults [9]. Despite the association with obesity, 40% of patients with MASLD are not obese, and 20% are lean [10], showing similar risks of disease progression as patients with obesity-related MASLD [9]. In this context, the role of healthy lifestyles based on diet and constant physical activity is crucial in the management of MASLD, being one of the triggers of and—modifiable—contributors to the progression of the disease to more severe conditions, but also offering treatment and prevention options [11,12]. Thus, the potential of dietary intervention in MASLD is significant, but implementation of and adherence to such interventions pose substantial challenges, constituting a significant hurdle [13,14]. Among various dietary strategies aimed at reducing and controlling the early stages of MASLD, the Mediterranean diet (MD) has been spotlighted as the reference diet [12]. Importantly, because of its focus on the intake of whole grains, fruits, vegetables and monounsaturated (MUFA) and polyunsaturated fatty acids (PUFA), the MD counters diets characterized by red meat, refined cereals, saturated and trans-fatty acids, well known as nutritional components that have deleterious effects on the MASLD risk [15,16]. The composition of the MD, in addition to weight loss *per se*, appears to be responsible for its benefits in reducing intrahepatic fat, oxidative stress and improved insulin sensitivity [15]. Moreover, a previous meta-analysis suggested that supplementation with omega-3 (n-3) fatty acids, abundant in the MD, may lead to reduced hepatic fat and contribute to these effects [17]. 

On the other hand, clinical studies have shown significant associations between diets high in animal fat, red and processed meats, sugar-sweetened beverages, and other sources of added sugars with an increased risk of hepatic steatosis [18], laying the foundation for the exploration of alternative dietary patterns [19]. In this sense, plant-based diets (PBDs), which are rich in fruits, vegetables, nuts, seeds, legumes, and whole grains, with less emphasis on animal and processed foods, constitute a useful and practical approach to chronic disease prevention [20]. However, while these dietary patterns have shown beneficial effects on metabolic syndrome (MetS) and MASLD, the hepatic manifestation of MetS [21], several concerns persist, including nutritional adequacy, particularly regarding protein and micronutrient intakes, and practicability, constituting contentious issues that are still debated [13,22]. 

Given the increasing prevalence of MASLD worldwide, and the growing evidence of different dietary patterns, it is essential to understand the role of PBDs in this condition. On that basis, this review aims to critically examine the impact of PBDs on MASLD, weighing up both the nutritional benefits and the potential health risks.

## 2. What Is a Plant-Based Diet?

A growing number of individuals are adopting PBDs [23]. Among the primary motivations for this shift is the pursuit of various health advantages, including the management of body weight [24]. The concept of PBDs has evolved significantly over the years, reflecting changes in nutritional, environmental, ethical and public health understandings, but also creating some confusion regarding its interpretation [25,26].

In this regard, the term PBDs has encompassed definitions that align with veganism, a regime that excludes any animal-based foods, as well as others that include the limited intake of foods of animal origin. For this reason, pending a consensus on the definition, a proposal to use the term PBDs only when accompanied by a detailed dietary description was put forward [25]. Based on the need for a standardized definition to improve clarity and consistency in research and public discourse, Hargreaves et al. proposed an updated way to understand a PBD as “a dietary pattern in which foods of animal origin are totally or predominantly excluded”, encompassing a pattern focusing on plant foods, reducing the intake of animal products, and including vegetarian and non-vegetarian diets (such as the MD and the dietary approaches to stop hypertension, among others), as long as the intake of animal products is low [26].

**Figure 1:**
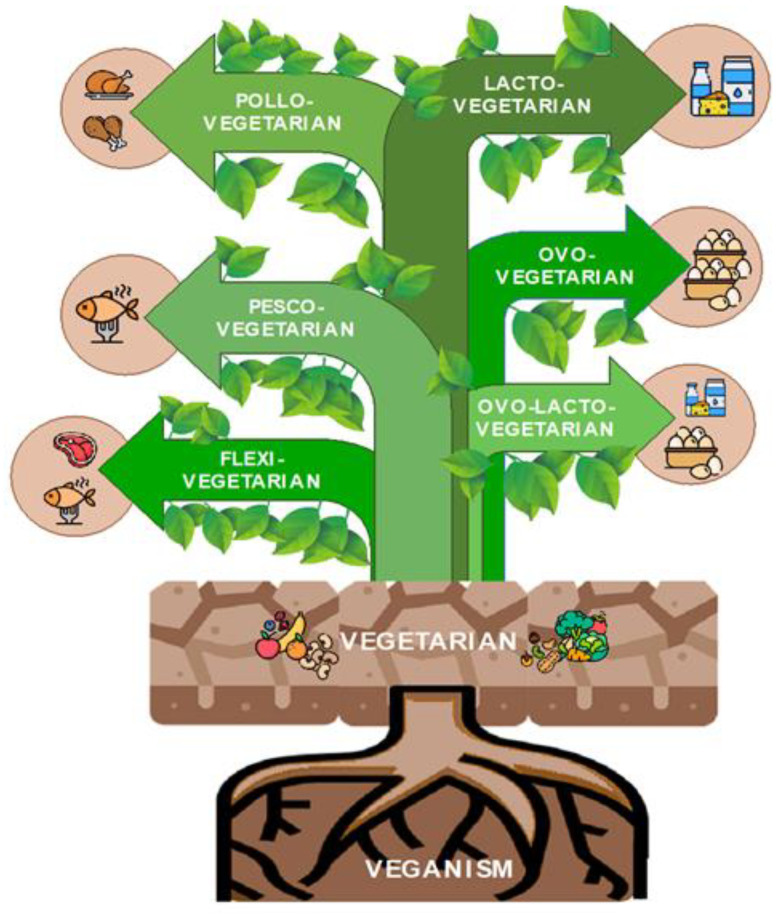
Classification of plant-based diets according to the consumed products. Within the vegetarian world, there are a number of nuances that suit different needs and personal beliefs: from the pesco-vegetarian, which includes fish consumption, to the pollo-vegetarian, which excludes red meats, the ovo-lacto-vegetarian, including eggs and dairy products, the lactovegetarian, excluding meat and eggs but embracing dairy products, and the ovo-vegetarian, which forgoes dairy products but includes eggs.

PBDs in fact comprises a rich variety of dietary approaches (Figure 1), including vegetarianism, which has historical roots and excludes meat and other animal products to varying degrees, and veganism, a stricter subset of vegetarianism that has emerged in recent times and excludes all animal products, but also lesser-known options such as the flexitarian, where meat intake is allowed. Whether these approaches fall into the category of PBDs depends on the ratio of animal to plant foods consumed, which is crucial in determining whether a diet should be classified as “high” or “low” in animal products [26]. Therefore, as long as animal products are limited in favor of plant-based ingredients, various diets can be considered part of the broad “plant-based” umbrella.

PBDs tend to be characterized by a high intake of foods that are rich in fiber, polyphenols, antioxidants and various nutrients, and their consumption has been inversely associated with the risk of chronic diseases and mortality. A meta-analyses of 95 cohort studies concluded that for every 200 g/day increment in fruits, vegetables, and combined fruits and vegetables intake, an 8–16% reduction in coronary heart disease, 13–18% in stroke, 8–13% in cardiovascular disease (CVD), 3–4% in total cancer and 10–15% in all-cause mortality was observed [27]. In addition, the low saturated and high unsaturated fats content of a healthy PBD may reduce the CVD risk by improving the blood lipid profile. In this context, it was found that diets based on high amounts of foods, such as whole grains, fruits, vegetables, nuts, legumes, oils, tea and coffee, tend to reduce the risk of CVD, while those that include high amounts of plant products, such as refined grains, fried potatoes and foods and drinks with added sugars, may increase this risk [28]. In this regard, an analysis of two regimes, one characterized by foods considered healthy and the other by less healthy ones, showed that the cardio-metabolic effects vary depending on the quality of the foods included, highlighting the importance of careful food selection in PBDs [29].

Importantly, the adoption of PBDs faces several barriers identified in high-income countries, summarized by themes such as financial, emotional, knowledge, health, social and accessibility, among others. Some widely recognized hurdles are the perceived high cost of PBDs, the limited knowledge of what constitutes them and the concern about meeting nutritional requirements without animal products. However, it is important to emphasize the importance of the social factor, i.e., the feeling of a lack of acceptance, support and fear of judgment, which are emotionally aggravating triggers for dietary changes. Furthermore, factors such as habit and the general lack of accessibility of various plant-based options, especially when eating out or “on the go”, complicate the transition to this diet, constituting significant obstacles [24,30,31]. Other factors that may also influence the switch to a PBD may be age and stage of life. Younger adults may adopt a PBD more readily due to health beliefs and social influences, while older adults may find the diet beneficial in treating age-related problems such as cognitive decline [32,33].

Thus, as the definition and understanding of PBDs continues to evolve, the emphasis remains on promoting a healthy, accessible and sustainable dietary pattern in line with individual preferences and global health goals.

## 3. Impact of Plant-Based Diets on MASLD Outcomes

The major determinants of MASLD include sedentary lifestyles and poor-quality diets rich in sugar intake, which result in a positive energy balance. Adherence to PBDs characterized by a high consumption of healthy plant foods is associated with lower risks of chronic disease mortality, including CVDs, and has been endorsed for improving overall health [34,35]. In a cohort of middle-aged adults (n = 12,168) from the Atherosclerosis Risk in Communities study, who were followed for 29 years, it was found that increased adherence to plant-based and pro-vegetarian diets significantly reduced the risks of CVD, cardiovascular mortality and all-cause mortality [36]. These dietary patterns are also associated with a lower prevalence of MASLD, reduced liver fat content and decreased risks of hypertension and T2DM [37]. However, even with this pattern, high consumption of sugary drinks, snacks and refined products was linked to an increased risk of these diseases, again emphasizing the importance of healthy choices [20]. 

A study with over 60,000 participants showed that individuals adhering to PBDs had a lower average body mass index (BMI) compared to those following omnivorous diets. The CARDIVEG study compared vegetarian and MD and found that both led to weight loss and BMI improvement, although the impact on lipid profiles was mixed, suggesting no clear superiority of the vegetarian diet over the Mediterranean approach [38]. Furthermore, the BMI, a surrogate marker of adiposity, has various limitations as it fails to consider body composition, fat distribution and other important health-related factors such as sex, age or ethnicity [39,40]. Further results of the CARDIVEG study on 30 healthy subjects assigned to a 3-month Mediterranean or lacto-ovo-vegetarian diet showed that high-density lipoprotein (HDL) activity in promoting cholesterol efflux was more effective in the MD group, making it potentially superior for cardiovascular prevention [41]. Another 16-week randomized controlled trial assessed the effects of a low-fat vegan diet vs. a control diet on overweight individuals. The initial results highlighted significant reductions in body weight (treatment effect −6.5 kg, *p* < 0.001), fat mass (treatment effect −4.3 kg, *p* < 0.001), and insulin resistance (treatment effect −1.0, *p* = 0.004) in the vegan group [42]. A subsequent year’s analysis revealed that a vegan diet low in saturated and trans fats in favor of PUFA further reduced the fat mass and improved insulin secretion [43]. Furthermore, a recent review analyzed randomized controlled trials demonstrating that PBDs, specifically vegan and lacto-vegetarian diets, benefit MetS components such as reduced body weight and waist circumference, along with improved lipid profiles, plasma glucose and blood pressure levels [44]. In a study involving 219 adults with a BMI between 28 and 40 kg/m^2^, participants were either assigned to follow a low-fat vegan diet for 16 weeks or make no changes to their diet. The results highlighted a significant association between weight loss and dietary changes, especially with increased consumption of legumes (r = −0.38; *p* < 0.0001) and decreased consumption of meat, including fish and poultry (r = +0.43; *p* < 0.0001) [45].

Importantly, only a few plant-based regimes include animal products in minority proportions. For this reason, a randomized crossover study of 30 middle-aged participants with MetS who followed a PBD for 13 weeks examined the effects of egg consumption as part of a PBD on disease biomarkers. Participants were randomized to include two eggs or an equivalent amount of egg substitute with spinach at breakfast for two four-week periods, separated by a three-week washout period. The findings revealed a significant reduction in body weight (*p* < 0.02) and improvements in HDL cholesterol (*p* < 0.025) and plasma zeaxanthin levels (*p* < 0.01) in the egg group compared to the egg substitute group, highlighting that the inclusion of eggs in a PBD could improve some cardiovascular risk biomarkers in subjects with MetS [46].

On the other hand, several studies and trials have explored the impact of PBDs on liver health (Table 1). The low-fat vegan diet has shown potential benefits in significantly reducing the hepatic lipid content by 34.4% (from 3.2% to 2.4%; *p* = 0.03), as assessed by proton magnetic resonance spectroscopy, and t this reduction was positively associated with the change in body weight and improved insulin sensitivity [47]. The extent of these health improvements can be attributed to the richness of plant-based foods in bioactive compounds, which could influence the potential development of MASLD, and their tendency to be lower in calories [48,49]. Further evidence supporting the benefits of healthy plant-based foods comes from a cross-sectional study of 3900 US adults, showing that the consumption of healthy plant foods was inversely associated with MASLD diagnosed with transient elastography (OR = 0.50, 95% CI 0.35, 0.72, *p*-trend < 0.001), whereas unhealthy plant foods intake was positively associated with MASLD (OR = 1.37, 95% CI 0.93, 2.02, *p*-trend = 0.009) even after adjustment for BMI (OR = 0.64, 95% CI 0.46, 0.87, *p*-trend = 0.006) [50]. In this regard, a research study from the 2005–2010 NHANES cohort involving 18,345 adult US participants shed light on the inverse relationship between a healthy PBD and the likelihood of MASLD as well as more favorable liver transaminase values [51]. These data find support from a longitudinal cohort study of 159,222 participants without MASLD from the UK Biobank, with a median follow-up of 9.5 years, where it was found that higher adherence to a healthy PBD was associated with lower liver fat content assessed by magnetic resonance imaging-proton density fat fraction (MRI-PDFF), compared to an unhealthy PBD. Furthermore, a PBD, especially a healthy one, was also associated with a lower risk of MASLD regardless of genetic susceptibility [37]. However, conflicting results were observed from a study on Buddhist priests compared to a matched control group, showing that a vegetarian diet does not protect against MASLD, thus questioning the protective hypothesis of vegetarianism against MASLD (29.9% vs. 25.05%, *p* = 0.055) [52]. 

Several studies have confirmed the role of specific macronutrients in the onset and development of MASLD, independently of the energy intake [53]. Macronutrients such as saturated fatty acids, trans fats, simple sugars and animal protein have been related to liver damage. Indeed, in the EU SWEET project, high consumption of sugar-sweetened beverages and low/no-calorie beverages were associated with MASLD prevalence (FLI-defined) [54]. Conversely, MUFA and PUFA (especially n-3 fats), dietary fibers and plant based proteins may be beneficial to the liver [48,55]. Regarding n-3 PUFA, their supplementation is often associated with improvements in the biochemical aspects of MASLD as well as a reduction in hepatic steatosis [56]. These beneficial effects of n-3 PUFA appear to be caused by the enrichment of plasma and hepatic lipids, where they exert the potential anti-inflammatory and/or anti-fibrotic effects described in experimental studies [57,58]. 

Dietary fiber intake, especially whole grains and vegetables, is associated with a lower risk of MASLD [59]. Fiber-rich foods promote satiety through increased chewing and regulate gut microbiome mechanisms by producing beneficial short-chain fatty acids [59,60,61]. Additionally, fiber reduces the overall fat and protein absorption by limiting the physical contact between nutrients and intestinal villi [62,63], leading to reduced digestible energy intake and contributing to weight management [63]. Another important mechanism by which dietary fiber, especially from oats, barley, and psyllium, may improve liver health is through the reduction of the total and low-density lipoprotein cholesterol levels [64]. 

Moreover, plant foods are rich in polyphenols, which produce nitric oxide, a key vasodilator for regulating blood pressure and the expression of genes related to the cardiovascular system [65,66,67]. In vitro studies showed that several dietary polyphenols can alter the absorption of sugars and carbohydrates, inhibit glucose and fructose transporters, and activate signaling pathways, such as AMPK, contributing to slowing down glucose absorption and reducing blood glucose levels [68,69,70]. Despite the limited clinical studies, cellular and animal evidence suggests that polyphenols, by modulating the AMPK/SIRT-1 axis, may have a positive influence on hepatic steatosis, mainly through regulation of adipokines and insulin sensitivity, reducing de novo lipogenesis and increasing β-oxidation of fatty acids [71]. 

All these mechanisms help explain the benefits of PBDs, including the reduced risk of mortality from chronic metabolic diseases and improved MASLD outcomes [72]. However, further research is needed to deepen our understanding of these relationships.

**Table 1:** Selected studies analyzing the impact of PBDs on MASLD and liver-related outcomes.

Authors—Year	Study Design	Hypothesis/Aim	Outcomes—Remarks
Choi S.H. et al., 2015 [52]	Cross-sectional and retrospective study comparing MASLD prevalence of 615 Buddhist priests and controls matched for age, sex, BMI and eventual MetS, who underwent routine health checks.	To assess the relationship between MASLD and vegetarian diets, taking into account MetS and obesity.	A vegetarian diet does not protect against MASLD. MASLD was significantly associated with male gender, WC, BMI, albumin, glucose, ALT, TG, low HDL and high LDL.
Mazidi M., Kengne A. 2018 [51]	Observational study in US adults from the 2005–2010 NHANES in 18,345 participants.	To investigate the influence of PDI, hPDI and uPDI on MASLD occurrence and liver function tests.	The results showed an inverse link between a healthy PBD and the likelihood of MASLD and more favorable liver function tests; whereas an unhealthy PBD would have the opposite effect.
Chiu T.H. et al., 2018 [73]	Cross-sectional study that included 2127 nonvegetarians and 1273 vegetarians who did not smoke or habitually drink alcohol and had no hepatitis B or hepatitis C.	To examine the association between vegetarian diets, principal food groups and MASLD, and then compare the extent of liver fibrosis between vegetarians and non-vegetarians in subjects with fatty liver.	A vegetarian diet resulted inversely related to fatty liver through reduced BMI. Substituting soy for meat/fish or whole grains for refined carbohydrates might be protective regardless of dietary pattern.
Kahleova H. et al., 2020 [47]	16-week RCT with 244 participants randomized to the low-fat vegan diet or to the control group with no dietary changes.	To measure the impact of a low-fat vegan diet on body weight, IR, postprandial metabolism, and intramyocellular and hepatocellular lipid levels in overweight adults.	A low-fat vegan diet effectively reduces body weight and increases metabolism after meals, probably due to increased insulin sensitivity from reduced hepatocellular and intramyocellular fats.
Chiarioni G. et al., 2021 [74]	Prospective, pilot study on 32 patients with MASLD who accepted to adhere to a 6-month vegan diet.	To explore the effect of a vegan diet on liver chemistry in a group of patients with MASLD.	Improved hepatic enzymes in MASLD patients on a vegan diet with the decrease in body weight being a variable that did not seem to be critical for the outcome.
Li X., 2022 [50]	NHANES 2017–2018 cross-sectional study of 3900 US adults.	To investigate through transient elastography the association between the overall PDI, hPDI and uPDI, and MASLD.	A healthful PBD was inversely related to the odds of MASLD, also after adjusting for BMI. Unhealthful PBDs, on the other hand, showed a positive association with MASLD.
Garousi N. et al., 2023 [75]	RCT with 75 overweight/obese adults with MASLD, randomly allocated to 3-month LOV-D or SWL-D groups.	To compare the effects of a LOV-D vs. a SWL-D on obese/overweight adults with MASLD.	Adherence to a 3-month LOV-D resulted in improved MASLD, anthropometric measures, glycemic-related markers and lipid profiles.
Lv Y. et al., 2023 [37]	Longitudinal cohort study on 159,222 participants in the UK Biobank.	To study the link between PDIs and MASLD risk and whether these associations might be modified by the genetic risk of MASLD.	Higher intake of healthful PBDs was linked to a lower MASLD risk and hepatic fat content calculated by MRI-PDFF, while unhealthful PBDs were linked to higher MASLD risk and intrahepatic steatosis.

Abbreviations: ALT: alanine transaminase; BMI: body mass index; hPDI: healthy plant-based diet index; HDL: high-density lipoprotein; IR: insulin resistance; LDL: low-density lipoprotein; LOV-D; lacto-ovo-vegetarian diet; MASLD: metabolic dysfunction-associated steatotic liver disease; MetS: metabolic syndrome; MRI-PDFF: magnetic resonance imaging—proton density fat fraction; NHANES: National Health and Nutrition Examination Survey; PBDs: plant-based diets; PDI: plant-based diet index; RCT: randomized controlled trial; SWL-D: standard weight-loss diet; TG: triglycerides; uPDI: unhealthy plant-based diet index and WC: waist circumference.

## 4. Implementation of a Plant-Based Diet in MASLD Management

The management of MASLD primarily involves caloric restriction through lifestyle modifications and increased physical activity [76]. The MD has been shown to be the most effective approach, but other dietary strategies, such as low-fat, low-carbohydrate, and ketogenic diets, have also shown positive effects on metabolic disease outcomes [12,19]. While the short-term results favor low-carb diets, both low-carb and low-fat diets are similarly effective long term, particularly in reducing hepatic fat and improving liver health, as long as significant weight loss is achieved [77,78]. Recent strategies like time-restricted eating are also gaining attention [79]. In this context, PBDs could offer promising alternative approaches, as they may reduce the risk of obesity, low-grade inflammation, and impaired insulin sensitivity [80]. The introduction of a healthy PBD, emphasizing whole grains, fruit, vegetables, nuts, legumes, tea, coffee and non-hydrogenated vegetable oils, could therefore also offer substantial benefits in the management and prevention of MASLD [13,81] due to the high dietary fiber, antioxidants and PUFA contents, which are known to improve insulin sensitivity and overall metabolic health [47,82]. The lower energy density of PBDs, primarily due to their lower saturated fat and high fiber content, can also help to reduce the overall caloric intake, potentially decreasing hepatic fat accumulation [73,83].

However, the implementation of PBDs must be cautious and personalized, especially considering the high carbohydrate content that could, in the presence of insulin resistance—a frequent condition in MASLD patients—lead to increased hepatic fat storage [84]. Of note, strict adherence to PBDs may result in deficiencies in nutrients such as n-3 fatty acids. In fact, in an intervention study, compared with the controls, individuals with MASLD had lower intakes of n-3 and higher intakes of the n-6/n-3 fatty acids ratio [85]. Indeed, in a systemic review and meta-analysis, it was suggested that n-3 PUFA supplementation may reduce hepatic fat, although the optimal dose is currently unknown [17]. In addition to n-3, strict adherence to PBDs might also lead to deficiencies in nutrients such as vitamin B12 and iron, requiring careful dietary planning and possible supplementation [86,87]. Approximately 50% of vegans have been shown to be deficient in vitamin B12, underlining the importance of careful diet planning to avoid adverse health outcomes [88]. For this reason, it is important to be able to identify B12 deficiency symptoms that include numbness, tingling in the hands and feet resulting in motor difficulties, decreased sensitivity, dementia, depression, general weakness and psychosis, as well as the need for careful planning of the diet and supplementation in vegan/vegetarian individuals. In this regard, fortifying plant-based foods with vitamin B12 can be an important step in preventing its deficiency, helping to fulfill the daily needs of people adhering to these types of diet [89]. Regular monitoring of nutrient levels and liver health parameters is also essential to adjust the diet as needed and prevent adverse effects. 

Moreover, PBDs are environmentally sustainable, offering lower greenhouse gas emissions and reduced natural resource use compared to meat-based diets [90]. This aspect can further motivate patients to adopt and stick with a plant-based approach, aligning personal health improvements with environmental benefits. Educational interventions and personalized nutritional guidance are key to successfully implementing a PBD in MASLD patients. Healthcare professionals, including dietitians and nutritionists, should provide continuous support to ensure that patients understand the benefits and limitations of a PBD, learn how to make healthful choices, and maintain long-term adherence.

In conclusion, while PBDs can be highly beneficial for MASLD management and are a more sustainable dietary option, they require careful implementation and monitoring to maximize the benefits and minimize the risks, particularly regarding nutrient deficiencies and dietary balance [91,92]. Just as “one-size does not fill all”, physicians must work together with patients to establish the most effective treatment plan tailored to each individual’s needs. Tailored dietary recommendations that consider individual dietary preferences and cultural backgrounds are crucial for enhancing adherence and achieving long-term health outcomes. Moreover, continued research is necessary to fully understand their long-term effects on various demographic groups and to refine dietary recommendations for MASLD management [93].

## 5. Conclusions

Plant-based diets offer potential benefits for weight control, risk reduction of coexisting conditions and improvement of metabolic health, making them relevant in managing MASLD. These diets are not unequivocally superior to the Mediterranean diet in terms of the clinical outcomes but may represent an alternative option due to their specific composition, making them especially relevant in addressing the complexities of metabolic diseases. We should emphasize that besides diet, other lifestyle factors such as adequate sleep, rest, and physical activity play a role in modifying the risk of chronic diseases. Future research should explore the direct effects of plant-based diets on MASLD and highlight the importance of tailoring these diets based on genetic studies and specific biomarkers to optimize their effectiveness in preventing and treating metabolic diseases.

## Data Availability

All data are publicly available.
